# Dietary exposure assessment of aluminium and cadmium from cocoa in relation to cocoa origin

**DOI:** 10.1371/journal.pone.0217990

**Published:** 2019-06-05

**Authors:** Carolin Fechner, Matthias Greiner, Helmut Heseker, Oliver Lindtner

**Affiliations:** 1 Exposure, German Federal Institute for Risk Assessment, Berlin, Berlin, Germany; 2 Institute for Food Quality and Food Safety, University of Veterinary Medicine Hannover, Foundation, Hannover, Lower Saxony, Germany; 3 Department of Sports and Health, Paderborn University, Paderborn, North Rhine-Westphalia, Germany; Università degli Studi di Milano, ITALY

## Abstract

Cocoa contains aluminium and cadmium as environmental contaminants while concentrations are supposed to be country of origin-related. Integrating origin in dietary exposure assessment could refine calculations. Averages or higher percentiles of concentrations in cocoa powder from German Food Monitoring (GFM) and cocoa consumption from the German National Nutrition Survey II (NVS II) were combined in standard scenarios. Additional origin-related scenarios used concentration data grouped into origin A (lower concentrations) and origin B (higher concentrations) as plausible origin information was rare. The most conservative standard scenario resulted in the highest intake estimates for aluminium and cadmium with 0.152 mg/week/kg BW and 0.363 μg/week/kg BW and covered the origin influence calculated in origin-related scenarios. Having plausible origin information would help to refine exposure assessment as it is exemplarily shown here that origin-related lower intake estimates are possible. Using the Eurostat database and the Mintel Global New Product Database (GNPD) generated more origin information for products available on the German market. For Germany, cocoa beans, cocoa powder and cocoa mass were mainly sourced in Côte d'Ivoire, while the Netherlands was the main distributor. Packages of cocoa powders were sourced from different origins.

## Introduction

Aluminium and cadmium are contaminants within the food supply chain and they are especially contained in higher mean concentrations in cocoa and cocoa-based products which is relevant for dietary exposure assessment [1–4].

There are indications that aluminium and cadmium concentrations in cocoa products are connected with the primary geographical origin of the cocoa beans [1, 5–11]. Highest aluminium concentrations are found in cocoa when investigating foodstuffs and a relation to provenance is discussed [9]. High aluminium concentrations in soils in Ghana reduce the growth of cocoa plants [10] and cocoa beans from Ghana had highest aluminium concentrations in comparison to Nigeria, Ecuador and Cameroon [11]. Cocoa products from South America mostly contain more cadmium than samples originated from Africa shown in several analyses [1, 5, 8, 11]. For example Ecuadorian soil is contaminated with cadmium, which is then also found in cocoa beans [6], but the cadmium content could be reduced during processing from cocoa beans to powder and mass [7]. Cocoa beans are supplied and processed globally to be added to different composite food items [7, 12–15]. In major steps of food supply chains, there are different influences on substances and finally on dietary exposure possible [16–18]. Local conditions in agricultural primary production could influence substance concentration in foods and dietary exposure, as well as factors like time, climate, contact materials and processing steps in transport, storage and further production. The labelling of the primary geographical origin as country of origin or place of provenance is legally required for some, mostly agricultural products, but not for processed products like cocoa powder [19–23]. The origin labelling of pork, sheep, goats and poultry meat was regulated in 2011 [19], while origin labelling of beef meat was established in 2000 [23]. European and national regulations on origin labelling exist for eggs, honey, wine, olive oil [21], fresh fruits and vegetables [20, 22], fish products, organic products and products with traditional production methods in a special geographical area [19]. In the case of cocoa powder, information on the producing country may just be available based on voluntary declarations and help to identify the primary geographical origin.

In this article the primary geographical origin, in short origin, of cocoa is considered in relation to dietary exposure to aluminium and cadmium and compared with standard exposure scenarios as it has already been done for some agricultural products [24]. Normally, origin-related exposure scenarios are not considered [25]. German data on aluminium and cadmium concentrations in cocoa powder and consumption of cocoa powder and cocoa mass is used. A refined approach for assessment strategies based on limited origin data is introduced. The exposure assessment focusses on chronic intakes. In particular, it is investigated whether conservative assumptions cover origin-specific scenarios.

## Materials and methods

### Chronic dietary exposure assessment for aluminium and cadmium from cocoa powder

Chronic dietary exposure assessment for aluminium and cadmium from cocoa powder was performed using a general exposure model as in a previous investigation on unprocessed agricultural products [24]. Distribution parameters of aluminium and cadmium concentrations in cocoa powder were used together with a consumption distribution of cocoa on an individual level to calculate eight exposure scenarios. Four deterministic standard scenarios are applied normally, where mean food consumption and mean substance concentration in food are combined in a basic scenario while three further scenarios take high consumption (using percentile 95 (P95)) or high concentration (P95) into account as well [25]. These standard scenarios were used as a basis and extended by four origin-related scenarios using grouped substance concentrations from German Food Monitoring (GFM) to compare standard exposure in scenario 1 to 4 with origin-related situations in scenario 5 to 8. Moreover, the food consumption on an individual level was used as a distribution to perform exposure calculations instead of using the distribution parameters mean and P95. In this way it was possible to derive percentile 50 (P50) and P95 exposure out of the consumption distribution calculating with mean and P95 concentrations. The scenarios are constructed as it follows. In standard scenarios 1 and 2, mean substance concentrations of *all samples* were used to calculate P50and P95 exposure, while in standard scenarios 3 and 4 P95 substance concentrations of *all samples* were used to calculate P50 and P95 exposure. In scenarios 5 and 6, mean substance concentrations from *origin A* (grouped lower concentrations) were used to calculate P50 and P95 exposure. In scenarios 7 and 8, mean substance concentrations from *origin B* (grouped higher concentrations) were used to calculate P50 and P95 exposure. A chronic consideration of substance intake made it possible to take a look at long-term influences. The calculated exposures for aluminium and cadmium from cocoa powder were compared with the tolerable weekly intake (TWI) which is fixed for aluminium at 1 mg/week/kg BW [2] and for cadmium at 2.5 μg/week/kg BW [3].

### Cocoa consumption

Representative consumption data was obtained from the dietary history interview of the German National Nutrition Survey II (NVS II) [26]. NVS II interviews started in November 2005 and were recorded during one year in 4 waves at different sampling points all over Germany to depict seasonal effects [27]. The dietary history was a retrospectively request over four weeks and documented the frequency and quantity of foods and beverages usually consumed by 15 371 participants aged between 14 and 80 years as a representative sample for the German population. To derive consumption amounts of cocoa powder and cocoa mass, a disaggregated data version which was created for the LExUKon project [28–30] was used. In this project, all available food items including industrial processed and composite ones were disaggregated to consumption amounts of single ingredients using Bundeslebensmittelschlüssel (BLS) recipes [28–30]. E.g. bread consumption was disaggregated to the ingredients flour, water, salt and yeast to derive the consumption of single food components [28]. This disaggregated data version of food consumption was used to estimate lead and cadmium exposure from food in general [29, 30]. In the current investigation, appropriate BLS codes for cocoa powder and cocoa mass were used to extract consumption amounts out of composite foods, which were mostly confectionery baked goods, milk products and chocolate. A clear distinction between cocoa powder and cocoa mass consumption was not possible because similar food items like different chocolates were mostly disaggregated to cocoa powder but sometimes also to cocoa mass depending on available information from BLS recipes during the previous disaggregation of composite foods for the LExUKon project [28]. Therefore, the information on average long-term consumption of cocoa powder and cocoa mass was summarised to cocoa as a sum for each individual. Only data of consumers of cocoa was taken for further investigations.

### Aluminium and cadmium concentrations in cocoa powder and origin relations

To derive aluminium and cadmium concentrations in cocoa powder in connection with information on the geographical origin, GFM data including projects between 2005 and 2015 was used [31–33]. Analyses of cocoa powder were available from 2008 and 2012. A distinction of cocoa powder by fat content was not made as most of the cocoa powder was named as weakly fat-reduced but in some cases also as strongly fat-reduced or just as cocoa powder lacking in more information. All samples were summarised and considered as cocoa powder in the following investigations.

The Food and Agriculture Organization of the United Nations (FAO) database on crop production offered a possibility to check the origin declarations for cocoa powder in GFM because cultivation areas of cocoa beans are displayed [34]. Data of the years 2005, 2008, 2012 and 2015 was requested, as the chosen monitoring period for cocoa powder was 2005 to 2015 and within this period cocoa powder was analysed in 2008 and 2012. If a continent instead of a specific country was given in GFM, FAO data was checked for countries within this continent as done in a previous investigation [24]. This is important because the annual GFM reports declare that especially the stated origin Germany does not necessarily correspond to the country of origin but to the place of processing or packaging [35].

Because plausible origin information for cocoa powder in GFM was rare, this could not be used to group substance concentrations by geographical origin. Therefore, a theoretical origin influence on substance concentrations was introduced to test the effect of higher values on statistical parameters within a sensitivity analysis [36, 37]. After sorting the substance concentrations, two different approaches (Grouping 1 and Grouping 2) were used to divide the concentrations into three segments, i.e. lower, middle and higher values. Grouping 1 was compiled by arranging the same number of values in every segment, while Grouping 2 was created using percentile 25 (P25) as highest limit within the first segment and percentile 75 (P75) as the lowest limit within the third segment. In both approaches, the first segment represents lowest concentrations and was assigned to *origin A*, while highest concentrations (segment three) were attributed to *origin B* to compare the mean concentrations of these subsets with mean concentrations of *all samples*. For Grouping 1 and 2, mean concentrations of *all samples*, *origin A* and *origin B* were tested for significant differences using SPSS version 21. Differences between Grouping 1 and 2 were not assessed. The application of the Kolmogorov-Smirnov test showed normal distributed aluminium concentrations for *all samples*, *origin A* and *origin B* for both, Grouping 1 and 2. In the case of cadmium, concentrations were not normal distributed. Finally, the non-parametric Kruskal-Wallis test in combination with the post-hoc Dunn-Bonferroni test were used for multiple mean comparisons between *all samples*, *origin A* and *origin B* with a significance level of p ≤ 0.05.

### Origin of cocoa products on the German market

The rare occurrence of plausible origin information for cocoa powder in GFM led to further origin analyses. The Eurostat database [38] gave information on trade relations between countries, while the software Warenstrom-Info [39] made it possible to request data automatically. Data for the years 2008, 2012 and 2015 was requested. The import amount of cocoa beans to Germany from the seven biggest suppliers and a sum of all documented partners was extracted. Additionally, import and export amounts of cocoa beans for the Netherlands were extracted, as this country was a large trade partner of Germany. The same consideration as for cocoa beans was carried out for cocoa powder and cocoa mass as a sum of processed products. FAO data on cocoa bean production was used to check if the considered cocoa items are directly delivered from producing countries to Germany [34].

The Mintel Global New Product Database (GNPD) [40] was requested for new product launches of cocoa powder in Germany between the start of the database in January 1996 and May 2018. For every product, packaging photos were checked for origin information. FAO data on cocoa bean production was used to check if the given information on the packaging corresponds with cultivation areas [34].

### Statistical analyses

Exposure calculations and statistical analyses of origin-related substance concentrations from GFM as well as cocoa consumption from NVS II were carried out using SPSS version 21. In the case of substance concentrations, standard deviation (SD) was only provided for groups of at least four samples and P95 was only calculated for groups of at least 20 samples. Generally, P95 was determined using PTILE within the CTABLES command. Microsoft Excel 2010 was used for the graphic depiction of the calculated exposure scenarios and for the analysis of information in the FAO database, the Eurostat database and the Mintel GNPD.

## Results and discussion

### Cocoa consumption and chronic dietary exposure assessment for aluminium and cadmium from cocoa powder

Consumption data for cocoa powder and cocoa mass derived from NVS II [26] is displayed in Table 1. Cocoa powder and cocoa mass summarised show a P50 cocoa consumption of 0.014 g/d/kg BW and a P95 cocoa consumption of 0.098 g/d/kg BW for consumers of such food items. This consumption data was used for exposure assessment on an individual level. The consideration of cocoa consumption based on disaggregated recipes makes it possible to integrate all cocoa-based recorded food items into the study.

**Table 1:** Cocoa consumption in Germany according to German National Nutrition Survey II (NVS II) dietary history interview–consumers only. N number of consumers as a subset of 15371 participants in NVS II dietary history interview (frequency and quantity of foods and beverages usually consumed over four weeks, participants aged between 14 and 80 years). Consumer characteristics: 48.8% male (age: 43.7 ± 17.3 years; BW: 83.3 ± 14.9 kg), 51.2% female (age: 45.0 ± 17.9 years; BW: 69.3 ± 14.4 kg). BW body weight, SD standard deviation, P50 percentile 50, P95 percentile 95. BLS codes: cocoa powder (not specified S7100??, in general S710000, slightly defatted S711000, strongly defatted S713000); cocoa mass (S790000).

Food product	Number of consumers	Consumption amount [g/day/kg BW]		
	N	Mean ± SD	P50	P95
Cocoa powder	12 115	0.023 ± 0.036	0.012	0.081
Cocoa mass	3 802	0.019 ± 0.061	0.006	0.070
Cocoa sum	12 482	0.028 ± 0.050	0.014	0.098

**Fig 1:**
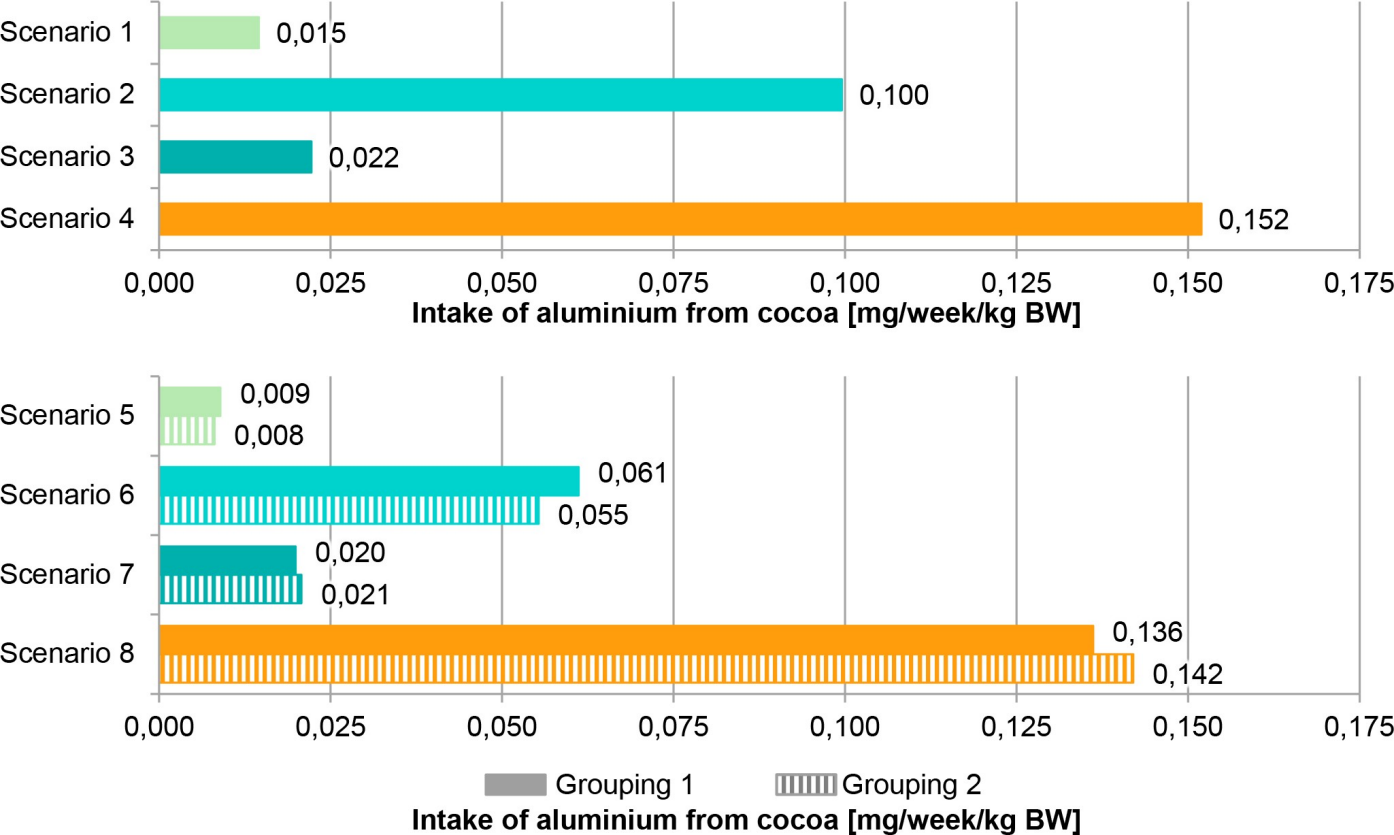
Chronic intake of aluminium from cocoa powder. Calculations based on German Food Monitoring (GFM) 2005–2015 and German National Nutrition Survey II (NVS II) dietary history interview. Same colour shows corresponding scenarios (standard (1–4) and origin-related (5–8)). While standard scenarios use percentile 95 (P95) for high concentrations, origin-related scenarios use mean concentrations of different origins. BW body weight.

**Fig 2:**
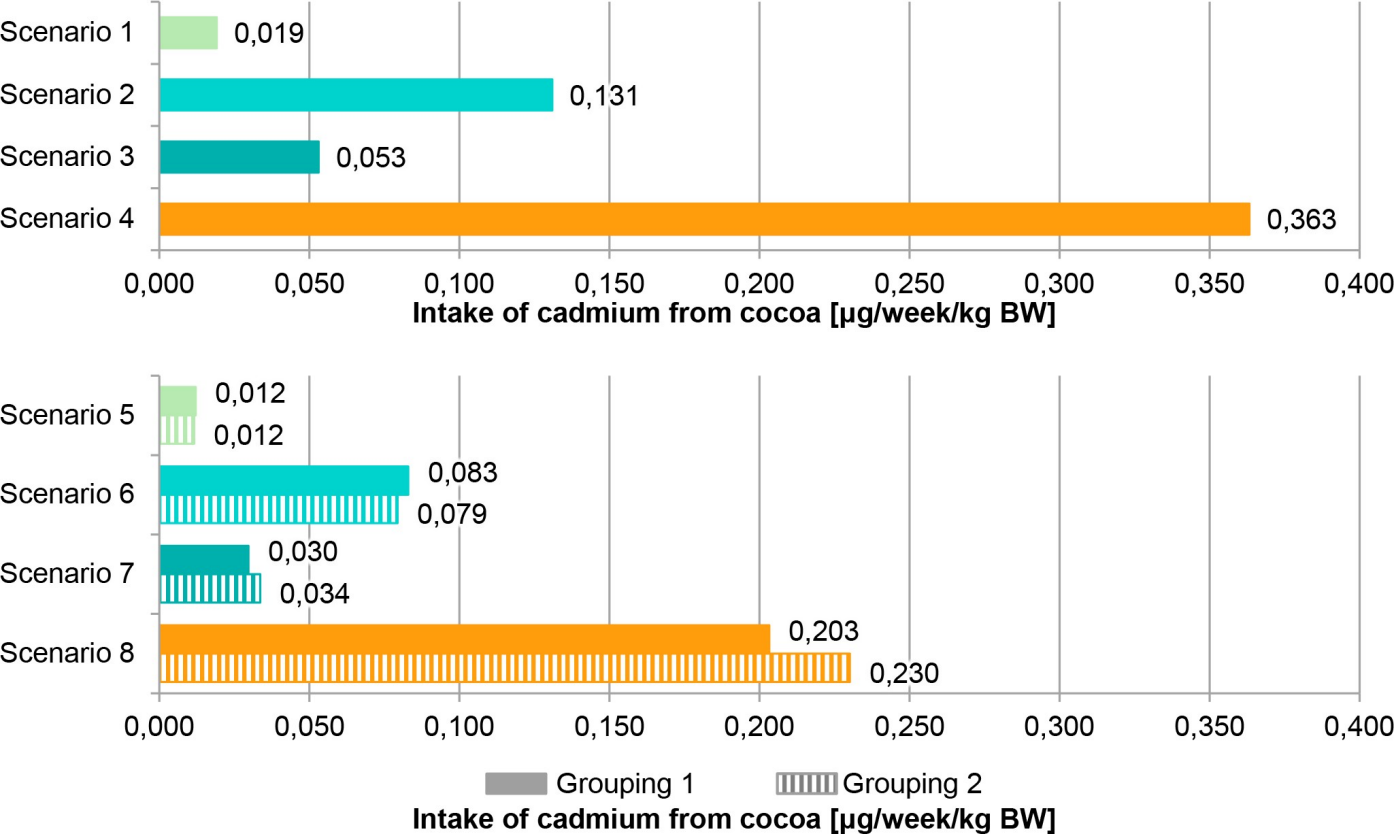
Chronic intake of cadmium from cocoa powder. Calculations based on German Food Monitoring (GFM) 2005–2015 and German National Nutrition Survey II (NVS II) dietary history interview. Same colour shows corresponding scenarios (standard (1–4) and origin-related (5–8)). While standard scenarios use percentile 95 (P95) for high concentrations, origin-related scenarios use mean concentrations of different origins. BW (body weight).

For chronic exposure assessment, it was assumed that higher aluminium and cadmium concentrations in cocoa powder are associated with a certain geographical origin, here *origin B*, and can have a relevant influence. A comparative consideration of standard exposure and origin-related intake linked to lower (*origin A*) and higher (*origin B*) aluminium and cadmium concentrations from cocoa powder used in various food items was done in Figs 1 and 2. The most conservative standard scenario 4 results in the highest intake estimates, 0.152 mg/week/kg BW for aluminium and 0.363 μg/week/kg BW for cadmium. Scenario 4 is based on the calculation with P95 concentrations of *all samples*, displays the P95 exposure and exceeded the most conservative origin-related scenario 8 which calculates with mean concentrations from *origin B* and displays P95 exposure. Scenario 8 resulted in 0.136 mg/week/kg BW (Grouping 1) and 0.142 mg/week/kg BW (Grouping 2) for aluminium, in the case of cadmium, it resulted in 0.203 μg/week/kg BW (Grouping 1) and 0.230 μg/week/kg BW (Grouping 2). Constructing a theoretical origin influence by grouping of concentrations shows an effect on exposure assessment. The two different grouping methods for aluminium and cadmium concentrations in cocoa powder showed slightly different intake estimates but values were in the same range. The different methodology performed for grouping did not have a strong influence on exposure assessment. The TWI is fixed for aluminium at 1 mg/week/kg BW [2] and for cadmium at 2.5 μg/week/kg BW [3]. The highest intake estimate (scenario 4) represented 15% of the TWI in the case of aluminium and also for cadmium, while the most conservative origin-related scenario 8 represented 14% of the TWI for aluminium and 8 – 9% of the TWI for cadmium (Grouping 1 and 2).

The results of exposure assessment indicate it is sufficient to focus on unspecific high concentrations (P95) of *all samples* considering chronic aluminium and cadmium exposure from cocoa powder because the exposure due to mean concentrations of origins with defined higher concentrations (*origin B*) is covered. But there is also a need to perform all standard scenarios to cover the possible origin influence because the intake estimate of origin-related scenario 8 exceeded the standard scenarios 1 to 3 (Figs 1 and 2). An additional focus on origin-related scenarios with defined concentrations from *origin A* and *origin B* could help to prevent overestimation of exposure and create refined approaches by the integration of origin-related aspects. Previous investigations also show the possibility that the origin-related scenario 8 represents the most conservative exposure estimate [24]. Exposure calculated from different scenarios shows the influence of assumptions and uncertainties in the approach. Focussing on only one food origin as a possible consumer behaviour is used as the base of origin-related scenarios to model a long-term consumption of lower (*origin A*) or higher substance concentrations (*origin B*). In this way, conscious consumer decisions for foods from specific regions, but also preferences of specific brands, varieties or selling points as unconscious influences on the choice of food origin, are integrated. The comparison of intake estimates derived from standard scenarios and origin-related scenarios shows a possible refinement in exposure assessment integrating data on food origin. More knowledge on consumer habits in relation to food origin is required for a refined construction of scenarios.

According to the EFSA, the total aluminium exposure from food and water for adults amounts to 0.2–1.5 mg/week/kg BW [2]. Results of the 2^nd^ French total diet study (TDS) show a mean total food exposure for adults of 0.28 mg/week/kg BW and a P95 exposure of 0.49 mg/week/kg BW, while the mean and the P95 exposure from chocolate amount to 1.18 μg/day/kg (i.e. 0.008 mg/week/kg BW) and 9.02 μg/day/kg BW (i.e. 0.063 mg/week/kg BW) [41]. The findings concerning aluminium in the current study (Fig 1) range between 0.008 mg/week/kg BW and 0.152 mg/week/kg BW. These results are in line with the other investigations as they are lower than the calculated total exposures and higher estimates are observed than for the aluminium exposure only from chocolate determined in the 2^nd^ French TDS. Cocoa consumption is a source of aluminium intake and contributes much to total dietary exposure. According to the EFSA, the total cadmium exposure from food for European adults amounts to 2.4 μg/week/kg BW in a mean consideration and 3.66 μg/week/kg BW in a P95 consideration [4]. Results of the 2^nd^ French TDS show a mean total food exposure for adults of 1.12 μg/week/kg BW and a P95 exposure of 1.89 μg/week/kg BW, while the mean and the P95 exposure from chocolate amount to 0.0021 μg/day/kg (i.e. 0.015 μg/week/kg BW) and 0.0159 μg/day/kg BW (i.e. 0.111 μg/week/kg BW) [41]. The findings concerning cadmium in the current study (Fig 2) range between 0.012 μg/week/kg BW and 0.363 μg/week/kg BW. These results are in line with the other investigations, as they are lower than the calculated total exposures and higher estimates are observed than for the cadmium exposure only from chocolate determined in the 2^nd^ French TDS. Cocoa consumption is a source of cadmium intake and contributes to total dietary exposure.

### Aluminium and cadmium concentrations in cocoa powder and origin relations

In GFM, 167 samples of cocoa powder were analysed for aluminium and 166 samples for cadmium between 2005 and 2015 (Table 2). Data for cocoa mass was not considered as fewer samples were available and concentrations were higher in cocoa powder. In this way, we followed a conservative approach. The distinction by origin declarations showed 12 cocoa powder samples with plausible origin information because cultivation areas according to FAO [34] were stated, i.e. regions in Africa, South America and Indonesia. Other origins in GFM were European countries and the Russian Federation, which are not classified as cultivation areas by FAO. Additionally, 26 not specified origin declarations appeared. A missing specification may be due to the fact that country of origin labelling is not mandatory for cocoa powder and only voluntary statements can be expected on the packaging [19]. Additionally, annual GFM reports declare that the stated origin Germany does not necessarily correspond to the country of origin or place of provenance but to the place of processing or packaging which was the case for cocoa powder in 2012 [35]. In this way, the country of origin is not defined detailed in GFM and processing or packaging stages could be named. This could be an explanation why European countries and the Russian Federation are stated in GFM as country of origin of cocoa powder.

**Table 2:** Aluminium and cadmium concentrations in cocoa powder by geographical origin available in German Food Monitoring (GFM) 2005–2015. N sample number, SD standard deviation. All cocoa powder samples analysed for aluminium and cadmium had quantifiable concentrations. Methods used for analysis: Graphite furnace atomic absorption spectroscopy (GFAAS) or Inductively coupled plasma mass spectrometry (ICP-MS). GFM codes: aluminium ss-1813000, cadmium ss-1848000, cocoa powder el-450401.

Geographical origin	Aluminium			Cadmium		
	N*	Mean [mg/kg]	SD [mg/kg]	N*	Mean [mg/kg]	SD [mg/kg]
European origins summarised	129	148	51	128	0.19	0.12
Austria	1	138	-	1	0.14	-
France, incl. Corsica	1	290	-	1	0.68	-
Germany	114	148	49	114	0.18	0.12
Italy	1	122	-	1	0.17	-
The Netherlands	6	147	69	6	0.15	0.04
Poland	1	51	-	1	0.13	-
Russian Federation	1	140	-	1	0.54	-
Switzerland	2	162	-	2	0.25	-
Spain	1	156	-	1	0.24	-
United Kingdom	1	166	-	-	-	-
African origins summarised	7	151	45	7	0.21	0.21
Africa ^a^	6	155	47	6	0.23	0.22
Côte d'Ivoire ^a^	1	130	-	1	0.08	-
American origins summarised	4	45	30	4	0.44	0.11
Bolivia ^a^	1	5	-	1	0.29	-
Dominican Republic ^a^	1	67	-	1	0.57	-
Peru ^a^	2	53	-	2	0.45	-
Indonesia ^a^	1	92	-	1	0.30	-
Not specified **	26	151	46	26	0.17	0.07

* For most of the geographical origins, sample numbers are low and statistically not significant.

** “without declaration”, “unexplained”, “unknown foreign country” summarised

^a^ Plausible primary geographical origin according to available FAO data on cocoa bean production in the years 2005, 2008, 2012 and 2015 [34]

Mean aluminium concentrations in cocoa powder ranged between 5 mg/kg and 290 mg/kg and mean cadmium concentrations ranged between 0.08 mg/kg and 0.68 mg/kg considering the original origin declarations in GFM (Table 2). For some origins very low sample numbers are available and in this way mean and SD cannot depict the possible variability of concentrations. Table 2 shows all origin information provided in GFM but the concentrations grouped by these origins are not used for further calculation. In this study, aluminium and cadmium concentrations in cocoa powder were considered for *all samples* and for subsets of *all samples* called *origin A* and *origin B* as the origin information in GFM was mostly not plausible and could not be used for origin-related grouping (Table 3). *Origin A* represents lower concentrations and *origin B* represents higher concentrations. For both, aluminium and cadmium, considering Grouping 1 and 2, there are statistically significant differences in mean substance concentrations between *all samples* and *origin B*, *all samples* and *origin A* as well as *origin B* and *origin A* (Table 3). Two further tested methods for the grouping of substance concentrations were discarded as the distribution of values into three segments was unequal using P10 and P90 for segmentation or dividing the scale of values into three equal parts.

**Table 3:** Aluminium and cadmium concentrations in cocoa powder sampled in German Food Monitoring (GFM) 2005–2015 grouped by theoretical concentration-related geographical origins. N sample number, SD standard deviation, P95 percentile 95. All cocoa powder samples analysed for aluminium and cadmium had quantifiable concentrations. Methods used for analysis: Graphite furnace atomic absorption spectroscopy (GFAAS) or Inductively coupled plasma mass spectrometry (ICP-MS). GFM codes: aluminium ss-1813000, cadmium ss-1848000, cocoa powder el-450401. Mean concentrations of all samples, origin A and origin B are compared. For both, aluminium and cadmium, considering Grouping 1 and 2, there are statistically significant differences in mean substance concentrations between all samples and *origin B*, all samples and *origin A* as well as *origin B* and *origin A* according to Kruskal-Wallis test (p ≤ 0.05).

Substance	Concentration groups *		N	Mean [mg/kg]	SD [mg/kg]	P95 [mg/kg]
Aluminium	All samples		167	146	52	223
	Grouping 1	*Origin A*	56	90	28	117
		*Origin B*	56	199	29	276
	Grouping 2	*Origin A*	42	81	28	110
		*Origin B*	42	208	29	276
Cadmium	All samples		166	0.19	0.13	0.53
	Grouping 1	*Origin A*	55	0.12	0.02	0.14
		*Origin B*	56	0.30	0.17	0.768
	Grouping 2	*Origin A*	42	0.12	0.02	0.13
		*Origin B*	42	0.34	0.19	0.68

* All samples: All available substance concentrations. Grouping 1: To group all available substance concentrations, sorted concentrations are arranged into three segments, each with the same number of values. Grouping 2: To group all available substance concentrations, sorted concentrations are arranged into three segments using percentile 25 and percentile 75. *Origin A*: Lower mean substance concentrations out of all samples related to a theoretical geographical origin (segment 1). *Origin B*: Higher mean substance concentrations out of all samples related to a theoretical geographical origin (segment 3).

Aluminium is an environmental contaminant and it is used in food additives and as packaging [2]. Analyses of cocoa powder show mean aluminium concentrations of 33 μg/g (i.e. 33 mg/kg) for samples bought in 1988 and 103 μg/g (i.e. 103 mg/kg) for samples bought in 1991, while most of the other investigated foodstuffs have concentrations below 5 μg/g (i.e. 5 mg/kg) [9]. Müller et al. concluded that the unknown origin of the sampled cocoa powder might be of importance for the significant concentration differences between the two investigated years. A mean aluminium concentration of 165 mg/kg is found in cocoa powder in another investigation, while the scientists remark that aluminium-containing additives are not allowed in cocoa powder and assume aluminium as a natural contamination in cocoa [42]. The reported aluminium concentrations in literature are within the range used for the current study (Table 2). Plants can absorb aluminium via their roots [43–45]. Aluminium-containing soils can retard the growth of cocoa seedlings and the mineral is more readily available for plants in acidic soils [46], which could be an explanation for higher concentrations from countries having such conditions. Especially in Ghana, the soils of cocoa plantations contain aluminium, which has an influence on cocoa plants [10]. Additionally, small scale mining on cocoa plantations is a common practice in Ghana because of the bauxite deposits [47] and could influence the aluminium concentration in cocoa beans. Next to local mining, there is a large aluminium industry located in West and Central Africa which is focussed on smelting aluminium from imported resources [48]. This could also influence the cocoa cultivation in Africa. Another item, which is discussed for Africa, addresses the production and usage of biochar to improve soil properties which could increase the aluminium content of the soil depending on the agricultural waste which is used for biochar production [49, 50]. A clearly higher mean aluminium concentration of 54 mg/kg for cocoa beans from Ghana than for the origins Nigeria, Cameroon and Ecuador, where the mean ranges between 9.2 mg/kg and 16 mg/kg, is shown [11]. This indicates that a summary of West African countries could cover information on aluminium variation in cocoa produced in this region. An investigation into larger regions shows significantly higher mean aluminium concentrations in cocoa beans from West Africa (155 mg/kg) and East Africa (274 mg/kg) than from Central America (41.1 mg/kg), while cocoa beans from South America and Asia are in between with 90.2 mg/kg and 89.1 mg/kg [51]. Available literature indicates higher aluminium concentrations are expected for products of African cocoa beans than from other regions. The reported Central American concentrations in cocoa beans are even lower than mean *origin A* concentrations in cocoa powder in the current study and mean aluminium concentrations for the summarised regions West and East Africa are in the range of the higher mean *origin B* concentrations (Table 3). This shows the importance of having origin information and integrating this data into evaluations for aluminium from cocoa powder. Constructing a theoretical origin influence by grouping of concentrations seems to be quite near to actual analysed aluminium concentrations in cocoa products from different geographical origins.

Considering cadmium, an analysis of cocoa products shows a decreasing mean concentration from cocoa powder via shells and beans to butter from 125 ng/g to < 3.1 ng/g (i.e. 0.125 mg/kg to < 0.003 mg/kg) and mean cadmium concentrations in four different chocolate brands containing 70% so-called cocoa solids, which might mean cocoa dry mass, ranged between 65 ng/g and 141 ng/g (i.e. 0.065 mg/kg and 0.141 mg/kg) [52]. These values are comparable to mean cadmium concentrations in cocoa powder from grouped *origin A* in the in current study (Table 3). Another work shows a median cadmium concentration of 0.116 mg/kg for chocolate with more than 50% dry mass of cocoa [53] which indicates that the cadmium concentration in chocolate comes mainly from cocoa, not from other ingredients. A median cadmium concentration of 0.159 mg/kg in cocoa powder [53] matches the findings here (Table 3). This is also in line with analyses of cocoa powders from two different producers which have a mean cadmium concentration of 0.153 mg/kg and 0.174 mg/kg, beans with shells are in the same range or higher, mass is lower and butter contains almost no cadmium [7]. The European Food Safety Authority (EFSA) shows a mean cadmium concentration of 183 μg/kg (i.e. 0.183 mg/kg) in cocoa powder as well [4]. A clearly higher mean cadmium concentration of 0.20 mg/kg for cocoa beans from Ecuador than for the origins Cameroon, Nigeria and Ghana, where the mean ranges between 0.05 mg/kg and 0.017 mg/kg, is shown [11]. This indicates higher cadmium concentrations are present in South American cocoa beans than in West African ones considering larger regions. An analysis of the Ecuadorian situation shows cocoa beans often contain more than 0.6 mg/kg cadmium ranging between 0.02 mg/kg and 3.00 mg/kg which is connected to high cadmium contents in soils due to anthropogenic influences [6]. An investigation on larger regions shows significantly higher mean cadmium concentrations in cocoa beans from South and Central America with 1388 μg/kg and 544 μg/kg (i.e. 1.388 mg/kg and 0.544 mg/kg) as well as from East Africa with 508 μg/kg (i.e. 0.508 mg/kg) and Asia with 328 μg/kg (i.e. 0.328 mg/kg) than from West Africa with the lowest mean of 92.6 μg/kg (i.e. 0.092 mg/kg) [51]. Another study shows the lowest mean cadmium concentrations in cocoa powder for Côte d'Ivoire and Ghana with 94 μg/kg and 133 μg/kg (i.e. 0.094 mg/kg and 0.133 mg/kg), while South American countries differ clearly as it is reported 125 μg/kg– 170 μg/kg (i.e. 0.125 mg/kg– 0.170 mg/kg) for two locations in Brazil, 533 μg/kg– 738 μg/kg (i.e. 0.533 mg/kg– 0.738 mg/kg) for two locations in Ecuador and 1833 μg/kg (i.e. 1.833 mg/kg) for Venezuela [8]. For Malaysia, a similar mean cadmium concentration of 602 μg/kg (i.e. 0.602 mg/kg) is given [8]. This shows a certain variation of cadmium concentrations in cocoa products from South America. A similar situation is shown for samples from the US market, as higher cadmium concentrations are shown for cocoa products with cocoa beans from Latin America than from Africa [5]. In comparison with grouped data of the current study, available literature indicates higher cadmium concentrations are expected for products of South and Central American cocoa beans, while West African cocoa beans are expected to have the lowest cadmium concentrations. The reported West African concentrations in cocoa beans would match mean *origin A* concentrations in cocoa powder in the current study and mean cadmium concentrations for the summarised regions of South and Central America as well as for East Africa even exceed the higher mean *origin B* concentrations here (Table 3). This shows the importance to have origin information and integrate this data in evaluations for cadmium from cocoa powder. But results of country-based analyses of cadmium concentrations also show a loss of information on variation by the consideration of summarised larger regions. Constructing a theoretical origin influence by grouping concentrations seems to be quite near to actually analysed cadmium concentrations in cocoa products from different geographical origins.

In GFM, analyses of aluminium and cadmium were available for the years 2008, and 2012 (Table 4). For aluminium, the mean concentration was higher in 2012 in comparison to 2008 but in both years it exceeded 100 mg/kg. This shows an existing contamination in both years, while the restriction to two investigation years and limited sample numbers do not allow the evaluation of aluminium concentrations over time. For cadmium, the mean concentration was 0.19 mg/kg in 2008 and 2012.

**Table 4:** Aluminium and cadmium concentrations in cocoa powder by years available in German Food Monitoring (GFM) 2005–2015. N sample number, SD standard deviation, P95 percentile 95. All cocoa powder samples analysed for aluminium and cadmium had quantifiable concentrations. GFM codes: aluminium ss-1813000, cadmium ss-1848000, cocoa powder el-450401.

Substance	Year	N	Mean [mg/kg]	SD [mg/kg]	P95 [mg/kg]
Aluminium	2008	80	118	51	231
	2012	87	171	38	216
Cadmium	2008	79	0.19	0.14	0.57
	2012	87	0.19	0.11	0.29

### Origin of cocoa products on the German market

Comprehensive plausible origin information was not available for aluminium and cadmium concentrations in cocoa powder in GFM and a theoretical origin influence was introduced to perform an origin-related exposure assessment. Additional origin information for cocoa beans (Table 5) and cocoa powder together with cocoa mass (Table 6) on the German market was obtained by analysing trade flows recorded in the Eurostat database [38]. The trade situation appeared generally stable for the considered years 2008, 2012 and 2015, as similar trade partners and volume are documented. Import data for Germany showed the Netherlands as a large supplier of cocoa beans and further processed cocoa products (Table 5, Table 6). Other European partners were also present but with an obvious lower trade volume. Germany was also directly supplied with cocoa beans, cocoa powder and cocoa mass from producing countries according to FAO data [34], mainly Côte d'Ivoire in Western Africa. This shows the processing of cocoa beans to cocoa powder and cocoa mass taking place in producing countries but also later within the complex supply chain. The Netherlands, as a large supplier of cocoa products to Germany, imported cocoa beans, cocoa powder and cocoa mass mainly from Côte d'Ivoire and other West African countries, so that most of the considered cocoa products arriving in Germany are from cultivation areas in Western Africa. The Netherlands clearly imported more cocoa beans than they exported (Table 5) and they imported less cocoa powder and cocoa mass than they exported (Table 6). This indicates that a great part of the imported cocoa beans is processed in the Netherlands and traded afterwards. The findings in the Eurostat database are supported by released data of the International Cocoa Organization (ICCO) which show Côte d'Ivoire and Ghana as the largest producers of cocoa beans and regarding the grinding as further processing step the Netherlands, Côte d'Ivoire and the USA have the highest amounts in the cultivation periods 2002–2007 and 2012–2014 [14, 54]. Besides all available information, data is also limited as Eurostat is dependent on correct trade reporting of all countries, small amounts of goods may be excluded, no direct information on goods reaching the customer is available and other trade channels like private importation of foods via internet cannot be regarded.

**Table 5:** Trade flows of cocoa beans for Germany and the Netherlands according to Eurostat in the years 2008, 2012 and 2015. Evaluations are based on the database Eurostat [38] using the software Warenstrom-Info [39]. HS code 18010000 “cocoa beans, whole or broken, raw or roasted” was considered.

Declarant	Trade flow	Year 2008			Year 2012			Year 2015		
		Partner	Amount [t]	Amount [%]	Partner	Amount [t]	Amount [%]	Partner	Amount [t]	Amount [%]
Germany	Import	Netherlands	82879	25	Côte d'Ivoire ^a^	109919	30	Netherlands	152217	38
		Côte d'Ivoire ^a^	80066	24	Netherlands	97123	26	Côte d'Ivoire ^a^	98538	25
		Togo ^a^	45571	14	Ghana ^a^	44319	12	Belgium	52773	13
		Nigeria ^a^	41848	13	Belgium	31645	9	Ghana ^a^	40525	10
		Ghana ^a^	20964	6	Nigeria ^a^	18523	5	Ecuador ^a^	11757	3
		Belgium	20826	6	Ecuador ^a^	15312	4	Guinea ^a^	6566	2
		Ecuador ^a^	13696	4	Luxembourg	13862	4	Uganda ^a^	5461	1
		*Sum all partners*	*334091*	*100*	*Sum all partners*	*369445*	*100*	*Sum all partners*	*397541*	*100*
Netherlands	Import	Côte d'Ivoire ^a^	230155	34	Côte d'Ivoire ^a^	234667	34	Côte d'Ivoire ^a^	278250	37
		Ghana ^a^	217645	32	Ghana ^a^	140556	21	Cameroon ^a^	126366	17
		Cameroon ^a^	127389	19	Cameroon ^a^	116591	17	Nigeria ^a^	119386	16
		Nigeria ^a^	62936	9	Nigeria ^a^	103238	15	Ghana ^a^	110572	15
		Togo ^a^	12275	2	Belgium	12060	2	Peru ^a^	22477	3
		Ecuador ^a^	6537	1	Dominican Republic ^a^	11704	2	Dominican Republic ^a^	18400	2
		Belgium	4804	1	Sierra Leone ^a^	11374	2	Ecuador ^a^	16493	2
		*Sum all partners*	*681032*	*100*	*Sum all partners*	*682449*	*100*	*Sum all partners*	*755261*	*100*
Netherlands	Export	Germany	74037	48	Germany	141200	78	Germany	179850	81
		Austria	19503	13	France	11527	6	France	11785	5
		France	16004	10	Belgium	11064	6	Belgium	7928	4
		United Kingdom	12345	8	Austria	5587	3	Italy	5816	3
		Belgium	12214	8	Italy	4804	3	Poland	3915	2
		Slovakia	7750	5	Greece	2843	2	Spain	3613	2
		Greece	3982	3	Spain	2374	1	Austria	3067	1
		*Sum all partners*	*155721*	*100*	*Sum all partners*	*181739*	*100*	*Sum all partners*	*221142*	*100*

^a^ Plausible primary geographical origin according to available FAO data on cocoa bean production in the years 2005, 2008, 2012 and 2015 [34]

**Table 6:** Trade flows of cocoa powder and cocoa mass for Germany and the Netherlands according to Eurostat in the years 2008, 2012 and 2015. Evaluations are based on the database Eurostat [38] using the software Warenstrom-Info [39]. HS code 18031000 “cocoa mass excl. defatted” and HS code 18050000 “cocoa powder, not containing added sugar or other sweetening matter” were summarised.

Declarant	Trade flow	Year 2008			Year 2012			Year 2015		
		Partner	Amount [t]	Amount [%]	Partner	Amount [t]	Amount [%]	Partner	Amount [t]	Amount [%]
Germany	Import	Netherlands	57429	59	Netherlands	61639	50	Netherlands	59201	49
		France	14187	15	Côte d'Ivoire ^a^	19271	16	Côte d'Ivoire ^a^	18217	15
		Côte d'Ivoire ^a^	5863	6	France	15993	13	France	14711	12
		Switzerland	4644	5	Ghana ^a^	13753	11	Ghana ^a^	12088	10
		Ghana ^a^	4295	4	Switzerland	4690	4	Switzerland	7876	7
		Austria	2342	2	Austria	2624	2	Austria	2868	2
		Belgium	2228	2	Ecuador ^a^	1239	1	Spain	1928	2
		*Sum all partners*	*97780*	*100*	*Sum all partners*	*123953*	*100*	*Sum all partners*	*120676*	*100*
Netherlands	Import	Côte d'Ivoire ^a^	54701	78	Côte d'Ivoire ^a^	63871	59	Côte d'Ivoire ^a^	72935	52
		France	5194	7	Ghana ^a^	23666	22	Ghana ^a^	33746	24
		Ghana ^a^	3304	5	France	4983	5	Germany	11185	8
		Germany	1889	3	Belgium	4935	5	France	11055	8
		Brazil ^a^	1225	2	Germany	4607	4	Belgium	5268	4
		Spain	635	1	Brazil ^a^	1715	2	USA	2139	2
		United Kingdom	569	1	Indonesia ^a^	1434	1	Indonesia ^a^	1344	1
		*Sum all partners*	*69683*	*100*	*Sum all partners*	*107945*	*100*	*Sum all partners*	*139839*	*100*
Netherlands	Export	Germany	66440	21	Germany	63478	18	Germany	61135	17
		USA	57204	18	USA	47623	14	USA	47973	13
		France	33294	11	France	37948	11	Belgium	35100	10
		Belgium	25668	8	Belgium	30727	9	France	30684	9
		Italy	17077	5	United Kingdom	18810	5	Italy	16552	5
		Poland	12261	4	Poland	17944	5	Turkey	15010	4
		United Kingdom	9950	3	Italy	15113	4	Poland	14208	4
		*Sum all partners*	*312480*	*100*	*Sum all partners*	*350270*	*100*	*Sum all partners*	*357156*	*100*

^a^ Plausible primary geographical origin according to available FAO data on cocoa bean production in the years 2005, 2008, 2012 and 2015 [34]

The Mintel GNPD [40] contains packaging photos of 14 cocoa powders launched between January 1996 and May 2018, eight were conventional and six were organic produced (Table 7). Two conventional products were from the summarised region West Africa but detailed reading of the whole product text was necessary to identify this information. Three organic products were labelled with non-EU and three further organic products had a country-specific origin labelling. All information given on geographical origins was plausible according to FAO data on cultivation areas [34]. But only the three country-specific labellings were highly specific and easy to find on the packaging. The declaration non-EU is plausible information but quite unspecific. For two conventional products, a further description of production steps in the Netherlands appeared, which was not a geographical origin information despite a country being stated. Every packaging carried an address of the importer, packager or producer which provided additional location data but not information on geographical origins. The new products listed since 1996 show that origin information is partly available but not standardised labelled as the declaration is not mandatory and only done voluntarily in terms of advertising [19]. This might be the reason for wrong origin reporting in GFM as processing stages are given instead of primary origin information (Table 2) [35]. The check of the Mintel GNPD helps to get an insight into the market situation but, as only new products are listed the information is limited.

**Table 7:** Labelled origin information for cocoa powder launched on the German market between January 1996 and May 2018 according to Mintel Global New Product Database (GNPD). N number of products listed with photos of packaging in Mintel GNPD [40].

Production	N	Place of origin declaration	Wording of origin declaration	Derived primary geographical origin
Conventional	5	-	-	-
	1	Product information	"Auswahl bester Bohnen aus Westafrika"	West Africa ^a^
	1	Product information	"aus feinem holländischen Kakaopulver [. . .] aus den besten Kakaobohnen Westafrikas"	West Africa ^a^
	1	Product information	"nederland kakao"	-
Organic	3	Information referring to EU organic logo	"Nicht-EU-Landwirtschaft"	Non-EU ^a^
	1	Explicit origin labelling	"Ursprungsland: Ecuador"	Ecuador ^a^
	1		"Herkunft Bio-Kakao: Dominikanische Republik / Südamerika"	Dominican Republic / South America ^a^
	1		"Ursprungsland: Indonesien"	Indonesia ^a^

^a^ Plausible primary geographical origin according to available FAO data on cocoa bean production in the years 2005, 2008, 2012 and 2015 [34]

### Conclusions

Origin-related sampling and testing of food is required for those substances that are known to have a geographical component in the prevalence of contamination or level of contamination. The case study on aluminium and cadmium in cocoa powder on the German market shows the need for a mandatory country of origin labelling for processed foods to be able to integrate origin relations into exposure assessment if there are hints of geographical connections. The highest intake estimates for aluminium and cadmium in this study represented already 15% of the appropriate TWI and origin influence should be considered in calculations in relation to human health. The performance of conservative standard scenarios without considering food origin may overestimate the dietary exposure in the case of aluminium and cadmium from cocoa powder. An underestimation is also possible if real origin relations are clearly different from the assumed theoretical conditions in the current study. With knowledge on food origin, dietary exposure estimates can be refined and more informative origin-specific scenarios can be used. Studies on consumer behaviour and surveys on industrial cocoa supply chains for the production of chocolate and other cocoa-containing foods could be additional valuable data sources.
